# CNN-Based Classification of *Ziziphus* Seeds with Focal Loss for Overcoming Size-Based Shortcut Learning

**DOI:** 10.3390/bios16060320

**Published:** 2026-06-02

**Authors:** Yea-Jin Park, Dae-Hyun Jung

**Affiliations:** 1 Department of Smart Farm Science, Kyung Hee University, Yongin 17104, Republic of Korea; lou0412@khu.ac.kr; 2 Interdisciplinary Program in IT-Bio Convergence System, Kyung Hee University, Yongin 17104, Republic of Korea

**Keywords:** herbal medicine, food safety, deep learning, image classification, loss function optimization, explainable artificial intelligence (XAI), shortcut learning

## Abstract

Herbal medicines represent a significant global market, yet food safety remains threatened by counterfeit products morphologically resembling authentic samples. Models trained on limited datasets are prone to shortcut learning, relying on superficial features rather than intrinsic morphological characteristics. This study identified size-based shortcut learning as a critical factor degrading the classification of *Ziziphus jujuba* Mill. var. *spinosa* and its counterfeit *Ziziphus mauritiana* Lam., and demonstrated that focal loss alone can effectively mitigate this issue. Models trained on the internal dataset were evaluated on an external dataset acquired with the Herb-X. On the internal test set, all configurations achieved high classification accuracies (≥98%), thereby obscuring meaningful differences in external generalization. However, consistent performance degradation was observed on the external dataset. The cross-entropy model trained on background-removed data dropped to 82.08 ± 10.97%, while size-normalized models recovered to 84.17 ± 10.15% (upsizing) and 88.94 ± 6.76% (downsizing), confirming that suppressing size shortcuts improves external generalization. The focal loss model, without any preprocessing, achieved 90.88 ± 2.71%, reducing the internal–external generalization gap from 16.18 to 8.11 percentage points. Grad-CAM++ and loss analyses confirmed that the focal loss model attended to intrinsic morphological features rather than object size. This study provides a practical, preprocessing-free approach for reliable herbal-medicine authentication in field conditions.

## 1. Introduction

Herbal medicines and traditional medicinal plants are vital resources utilized by approximately 80% of the global population for health maintenance and disease treatment [1]. The global market, valued at $165.13 billion in 2023, is projected to expand to $386.07 billion by 2032 with an annual growth rate of 11.20% [2]. However, increasing demand has led to various food safety problems, including the adulteration of visually similar species, counterfeiting, and inadequate quality control. Notably, reports indicate that the counterfeiting rate of herbal medicines reaches 21–80% in certain regions [3,4], severely threatening consumer safety and food security in the marketplace.

*Ziziphus jujuba* Mill. var. *spinosa* (Z. *jujuba*) of the Rhamnaceae family is an important herbal medicine used for various therapeutic purposes in traditional medicine [5]. Recent evidence demonstrates that extracts of Z. *jujuba* effectively reduce blood glucose levels [6], which has driven growing interest and increased demand. Consequently, the counterfeit product *Ziziphus mauritiana* Lam. (Z. *mauritiana*), which is morphologically highly similar, is frequently roasted and fraudulently distributed as Z. *jujuba* [5].

To address these issues, the Ministry of Food and Drug Safety conducts mandatory sensory evaluation of imported herbal medicines. Sensory evaluation exhibits low reproducibility due to high dependence on experts and faces challenges from increasing herbal medicine imports, the aging of sensory evaluation experts, and difficulties in training successive generations. Complementary methods, such as genetic testing and chemical profiling techniques like Thin-Layer Chromatography (TLC), are objective but possess limitations of being destructive and costly [7,8]. Thus, the need for non-destructive, rapid, and cost-effective methods for distinguishing authentic herbal medicines has emerged. Recently, attempts have been made to address these challenges by applying artificial intelligence (AI) methodologies to the classification of herbal medicines and plants. AI refers to technologies that mimic human learning capabilities and intelligence [9]. Deep learning is a machine learning technology that automatically learns complex patterns from large-scale data by stacking artificial neural networks into multiple deep layers [10]. Particularly, convolutional neural networks (CNNs) are representative deep learning models demonstrating powerful performance in computer vision, extracting features through filters and learning spatial information [11].

Recent advances in computer vision have enabled the widespread application of deep learning techniques to the classification of herbal medicines and medicinal plants, with RGB image-based approaches achieving high classification accuracy [12,13,14,15]. However, most existing studies evaluate these models using single datasets collected under controlled imaging conditions, limiting the validation of generalization performance in real-world environments [16]. Indeed, even in studies reporting high accuracy, variations in imaging conditions, such as lighting, acquisition devices, and sample states, have been identified as factors that may significantly affect model performance [14]. This suggests that high accuracy achieved under controlled experimental settings may not directly translate to real-world scenarios.

To address this limitation, we evaluated the model using an external dataset collected under different environments and observed that classification performance varied consistently with object size. Notably, higher accuracy was obtained when relatively larger samples were included in certain classes, whereas performance significantly degraded when the size distribution differed across datasets. These findings strongly indicate that the model relies on simple and easily exploitable cues, such as object size, rather than learning intrinsic morphological or structural characteristics of herbal materials. Furthermore, the consistent variation in performance with respect to size distribution across datasets suggests that object size may act as a primary decision criterion.

Based on these observations, we hypothesize that the model exhibits size-based shortcut learning, wherein it overly depends on dataset-specific features [17,18]. Such size-dependent decision making poses a critical challenge beyond technical instability, particularly in terms of practical deployment. Herbal medicines, as natural products, inherently exhibit variability in object size, which can further change depending on imaging conditions. Consequently, models relying on size are prone to significant performance degradation when applied to different datasets [19,20,21]. Furthermore, in the distribution of herbal medicines, counterfeiters often adopt strategies to evade authentication, including deliberately selecting or adjusting samples to mimic the size distribution of authentic materials [22]. Therefore, models that depend on object size are vulnerable to misclassification under such conditions, rendering size an unreliable discriminative feature.

To further investigate the underlying cause of this issue, we reviewed related literature and found that shortcut learning and spurious correlations have been widely recognized as critical challenges in computer vision and medical imaging domains [23]. Similarly, shortcut effects induced by non-causal features, such as background artifacts, have been reported in plant-related datasets [24]. These findings indicate that models relying on non-intrinsic features are inherently vulnerable to distribution shifts, which is closely aligned with the size-dependent performance variation observed in this study. In addition, prior work has shown that size-related preprocessing can influence model performance [25], further suggesting the impact of non-intrinsic visual cues. However, such issues have rarely been explicitly investigated in the context of herbal medicine classification, and there is limited evidence that size-based shortcut learning has been systematically analyzed or validated under distribution shifts.

To address size-based shortcut learning in this context, we propose loss function optimization as the intervention strategy. Among possible candidates, focal loss is a particularly natural choice. Although originally proposed for class imbalance in dense object detection [26], its core mechanism—down-weighting the gradient of well-classified samples and amplifying the training signal of hard samples—has recently been shown to mitigate spurious-correlation bias more broadly than its original class-imbalance application [27]. This suggests that focal loss may also be effective against size-based shortcut learning, motivating the loss-function intervention investigated in this study.

Therefore, this study aims to systematically investigate the presence of size-based shortcut learning in herbal medicine image classification and to develop a robust model that mitigates this issue by focusing on intrinsic morphological features rather than object size. To this end, we conduct comparative experiments across datasets with varying size distributions to verify the existence of shortcut learning. We further apply loss function optimization to reduce reliance on non-intrinsic features and employ explainable artificial intelligence techniques to analyze whether the model attends to meaningful object regions. Finally, we evaluate the generalization performance of the proposed approach using datasets collected under real-world conditions, thereby demonstrating its practical applicability.

## 2. Materials and Methods

**Figure 1:**
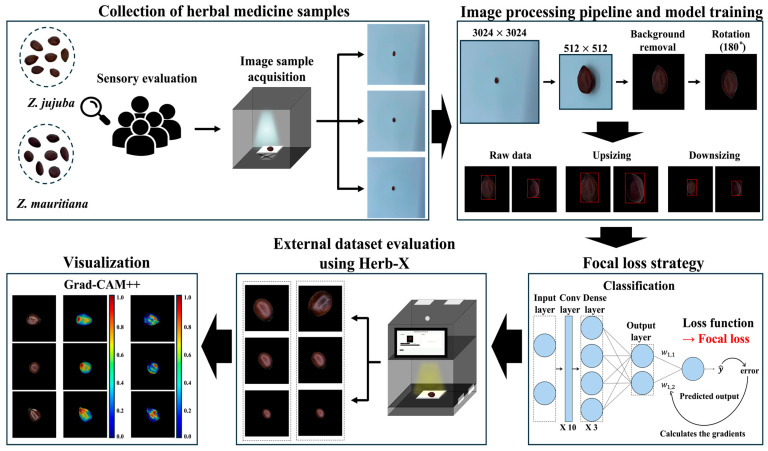
Overall workflow for herbal medicine classification. Red boxes indicate object size; the arrow shows the overall flow of the study.

The overall workflow of this study is presented in Figure 1, encompassing image acquisition, data preprocessing, model training, development of an explainable Herb-X, model performance evaluation, and visualization through Grad-CAM++.

### 2.1. Sample Preparation and Acquisition of Internal and External Datasets

Two independent image datasets were collected for this study. The first dataset, referred to as the internal dataset, was obtained from herbal medicine samples provided by the National Institute for Korean Medicine Development (NIKOM). Images were captured at 3024 × 3024 pixels resolution using an iPhone 8 (Apple Inc., Cupertino, CA, USA) and a Samsung Galaxy Note 10 (Samsung Inc., Suwon, Republic of Korea) within a PULUZ photography light chamber equipped with 25-lumen illumination and a white background.

**Figure 2:**
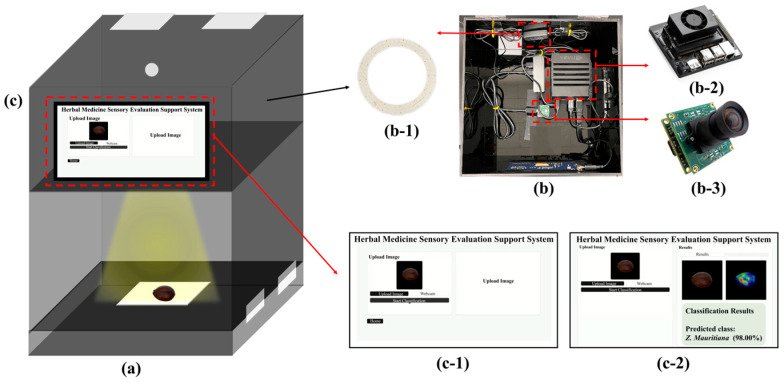
Herb-X for field-based sensory evaluation support: (**a**) Overall view of the chamber; (**b**) Key components of the chamber: (**b-1**) LED lighting strips, (**b-2**) NVIDIA Jetson Orin nano computer, (**b-3**) See3CAM_CU135_CHL_TC camera module; (**c**) LCD screen showing image acquisition and classification results: (**c-1**) Real-time imaging interface, (**c-2**) Herbal medicine classification and XAI visualization interface.

The second dataset, referred to as the external dataset, was acquired to evaluate classification performance under practical field conditions. This dataset was obtained using the Herb-X (Figure 2), which was fabricated to assist in the on-site sensory evaluation of herbal medicines. The chamber, measuring 300 mm × 300 mm × 320 mm, integrates in its upper compartment a compact computing unit (NVIDIA Jetson Orin, NVIDIA Corporation, Santa Clara, CA, USA) capable of real-time classification between counterfeit and authentic herbal medicines, 26-lumen illumination, and a liquid crystal display (LCD) for visualization. The lower compartment comprises a See3CAM_CU135_CHL_TC camera module (e-con Systems, Chennai, India) for image acquisition and a designated space for specimen placement during the imaging procedure. When a herbal medicine specimen is placed in the designated area and imaging is initiated, both the authentication results and Grad-CAM++ visualizations are displayed on the LCD screen.

**Figure 3:**
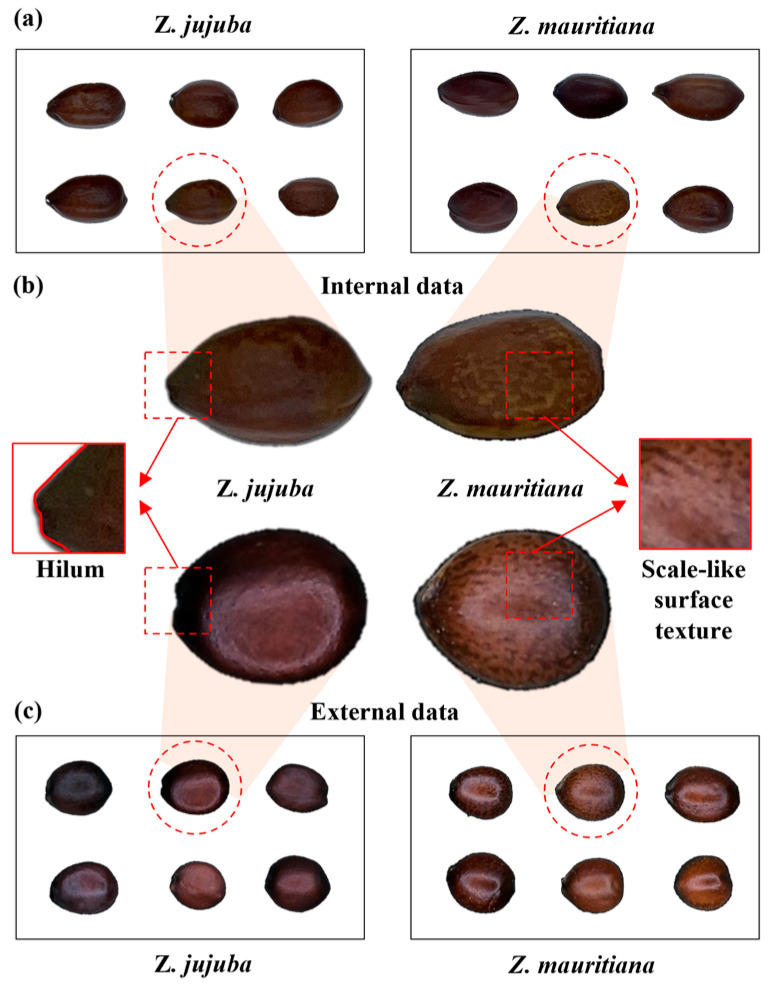
Representative images of *Z. jujuba* and *Z. mauritiana* seeds: (**a**) Representative images from the internal training set; (**b**) magnified views highlighting the key discriminative morphological features described in the main text; (**c**) representative images from the external test set acquired using the Herb-X.

The two datasets were collected from entirely independent sources, differing in geographic origin and harvest period, resulting in observable differences in specimen size, color, and morphology (Figure 3). All samples underwent verification by nine qualified sensory evaluation experts commissioned by the Ministry of Food and Drug Safety to confirm that they matched the accurate scientific names after collection. Although *Z. jujuba* and *Z. mauritiana* share broadly similar appearances and are frequently confused in commercial distribution, the two species exhibit subtle yet consistent morphological distinctions that form the biological basis for species-level discrimination (Figure 3). *Z. jujuba* seeds are reddish-purple to purplish-brown, with a smooth and glossy surface, and present raised ridge structures along the seed body and a concave, linear hilum at one end. In contrast, *Z. mauritiana* seeds are rounder and flatter, and exhibit a scale-like surface pattern.

### 2.2. Image Preprocessing and Size Normalization Strategy for Size-Based Shortcut Learning Mitigation

#### 2.2.1. Image Preprocessing and Data Augmentation

**Figure 4:**
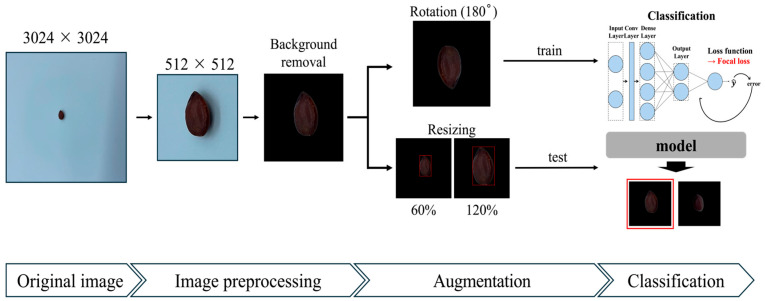
Data preprocessing and augmentation pipeline for training and test datasets. Red boxes indicate object size.

The collected original images at 3024 × 3024 pixels resolution were cropped to 512 × 512 pixels resolution, centered on the objects. Subsequently, the background was removed using the rembg library and overlaid on a black background. Thereafter, a 180° rotation augmentation technique was applied to ensure dataset diversity. The final dataset after these preprocessing and augmentation procedures consisted of 425 images of *Z. jujuba* and 392 images of *Z. mauritiana*. To verify the model’s robustness against physical size variations, the test dataset was generated with three different scale variations in original size (100%), reduced size (60%), and enlarged size (120%) (Figure 4).

#### 2.2.2. Size Normalization Strategy for Size-Invariant Dataset

Prior to the main experiment, a preliminary study was conducted to verify whether size-based shortcut learning occurs in herbal medicine image classification. First, object regions were detected in all images of the background-removed dataset to establish bounding boxes. Analysis of bounding box pixel areas revealed considerable size differences between *Z. jujuba* and *Z. mauritiana* objects.

Subsequently, to verify the effect of object size on model classification performance, new datasets were constructed using two normalization strategies that standardized object sizes within the dataset to control the object size variable. The first methodology, designated as “Upsizing,” standardized all bounding box sizes based on the largest object among the bounding box sizes of the two classes. Through this procedure, an image dataset consisting of 425 *Z. jujuba* and 392 *Z. mauritiana* images with identical object sizes was constructed, thereby eliminating dimensional disparities between objects. The second methodology, designated as “Downsizing”, proportionally reduced larger objects so that all bounding boxes aligned with the smallest bounding box size. This was a dataset that eliminated size differences between objects but increased class imbalance between objects and the background. The resulting dataset also consisted of 425 *Z. jujuba* and 392 *Z. mauritiana* images with identical object sizes across the two classes. In both methodologies, the aspect ratio of each object was strictly preserved to maintain morphological integrity.

### 2.3. Model Architecture and Loss Optimization Strategy for Size-Based Shortcut Learning Mitigation

#### 2.3.1. Deep Learning Model Architecture

**Figure 5:**
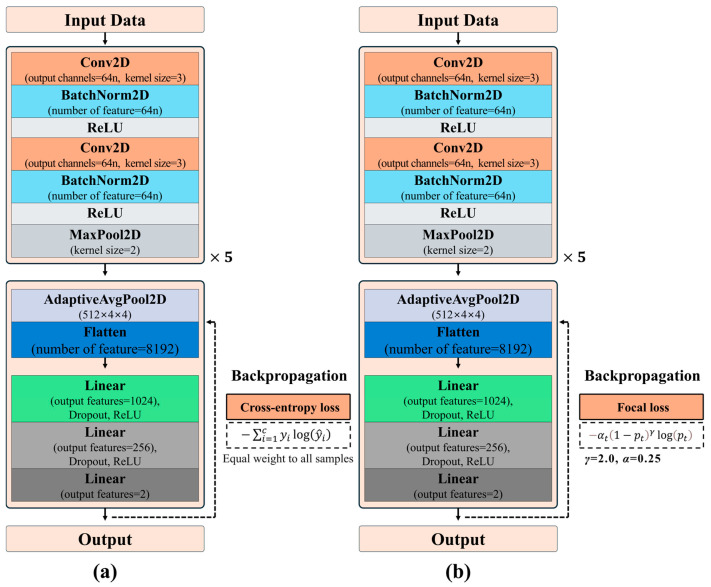
Deep learning model architectures: (**a**) Baseline model with standard cross-entropy loss; (**b**) Proposed model with cross-entropy loss replaced by focal loss.

To verify the effectiveness of focal loss in mitigating size-based shortcut learning in herbal medicine image classification, a CNN architecture was designed to receive 512 × 512 RGB images as input. The model consisted of five convolutional blocks with progressively increasing filter sizes (64, 128, 256, 512, and 512), where each block comprised two Conv2D layers with 3 × 3 kernels, batch normalization, ReLU activation, and 2 × 2 max pooling for spatial downsampling. After the convolutional blocks, adaptive average pooling reduced the feature maps to 4 × 4 spatial dimensions, followed by a flatten layer producing 8192-dimensional features and three fully connected layers with 1024, 256, and 2 neurons, respectively, incorporating dropout regularization rates of 0.5 and 0.3 for binary classification (Figure 5).

To verify that the conclusions drawn from this baseline generalize beyond the chosen architecture, ResNet-50 [28] was additionally evaluated as a representative, deeper, pre-trained backbone. ResNet-50 was implemented based on the torchvision.models.resnet50 module and initialized with the ImageNet1K_V2 pretrained weights (top-1 accuracy 80.86%). The final fully connected layer was replaced with a binary classification head (in_features = 2048, out_features = 2). The same set of dataset configurations (background-removed, Upsizing, Downsizing) and loss functions (cross-entropy, focal loss) used for the custom CNN was identically applied to ResNet-50.

#### 2.3.2. Deep Learning Model with Cross-Entropy Loss

For the CNN model prior to loss function optimization, cross-entropy loss, which is commonly used in general classification tasks, was adopted as the loss function. Cross-entropy loss, expressed as Equation (1), is a loss function that equally penalizes all samples based on the divergence between predicted probabilities and actual class labels and is most used in classification problems.

(1) $$L=-\sum_{i=1}^{c}y_{i}\log\left({\hat{y}}_{i}\right)$$

To verify the actual effect of object size on classification performance, performance was compared after training a CNN model applying cross-entropy loss on the background-removed dataset with object size differences and two size-normalized datasets. To verify that the observed effects are not specific to the custom 5-block CNN architecture, the same training procedure was independently applied to ResNet-50 across the identical three dataset configurations.

To ensure the statistical reliability of all reported metrics, every condition was repeated under 5-fold cross-validation crossed with 5 random seeds, yielding 25 independent runs per condition. The Adam optimizer and 50 epochs of training were applied. Learning rates were 4 × 10^−4^ for the 5-block CNN and 5 × 10^−4^ for ResNet-50. For each run, the best checkpoint was selected by validation accuracy. The category labels for *Z. jujuba* and *Z. mauritiana* were designated as 0 and 1, respectively.

#### 2.3.3. Loss Function Optimization with Focal Loss

To effectively mitigate size-based shortcut learning, the architectural structure of the baseline CNN was maintained by replacing the existing cross-entropy loss with focal loss. Focal loss is defined as follows in Equation (2).

(2) $$Focal\;loss=-\alpha_{t}{\left(1-p_{t}\right)}^{\gamma }\log\left(p_{t}\right)$$

p_t_ represents the predicted probability of the correct class, α denotes a weighting factor that controls class imbalance, and γ is a focusing parameter that modulates the degree of emphasis on hard examples. In this study, comparative experiments were conducted by configuring α to 0.15, 0.20, 0.25, 0.30, 0.40, 0.50, and 0.75 to determine the optimal performance of focal loss. Among α ∈ {0.15, 0.20, 0.25, 0.30, 0.40, 0.50, 0.75}, α = 0.25 yielded the best validation accuracy for the 5-block CNN and α = 0.50 for ResNet-50, and these values were used for the final models. The γ value was fixed at 2.0, as recommended in previous studies [26]. The background-removed dataset without size normalization was trained on the CNN model applying focal loss, and performance was compared with the CNN model applying the existing cross-entropy loss. The same procedure was independently applied to ResNet-50.

The rationale for applying focal loss to mitigate size-based shortcut learning, rather than to address class imbalance for which it was originally proposed [26], is grounded in the mechanism of its modulating factor ${\left(1-p_{t}\right)}^{\gamma }$. This factor reweights the per-sample training signal so that well-classified (easy) samples receive a small ${\left(1-p_{t}\right)}^{\gamma }$ weight and contribute little to the gradient, whereas misclassified or low-confidence (hard) samples retain large weights and dominate the gradient. When a model exploits a size-based shortcut, samples whose object size aligns with the dataset’s class–size correlation become “easy” and dominate learning, while samples that violate this correlation, namely those that can only be resolved through intrinsic morphological features, remain “hard”. Down-weighting the easy samples, therefore, implicitly redistributes the training signal away from the size, encouraging the model to learn intrinsic morphological features that generalize beyond the size cue.

#### 2.3.4. Baseline Methods for Comparison

To verify that focal loss is not merely outperforming a vanilla cross-entropy baseline but also outperforming alternative interventions commonly used to address generalization or class-imbalance issues, three additional baseline methods were evaluated on the 5-block CNN architecture under the same training protocol.

The first baseline was Class-Balanced Loss [29], a re-weighting scheme that assigns sample weights based on the effective number of samples per class to address class imbalance. The hyperparameter β, which controls the effective-sample re-weighting, was set to 0.9999 following Cui et al. [29]. The second baseline was Label Smoothing [30], a regularization technique that softens hard one-hot labels by distributing a small probability mass ε across the non-target classes, thereby preventing over-confident predictions. The smoothing factor ε was set to 0.1, implemented through the label_smoothing argument of nn.CrossEntropyLoss. The third baseline was Scale Jittering, a data-augmentation strategy in which each training image is rescaled by a random factor sampled at every training iteration, exposing the model to a wider range of object sizes and thus reducing reliance on absolute size cues. The scale factor was uniformly sampled from the range [0.8, 1.2] and applied only during training.

All three baselines were trained on the background-removed dataset. The Adam optimizer with weight decay 1 × 10^−4^, a batch size of 16, a cosine-annealing learning-rate schedule, 50 epochs of training, and a learning rate of 4 × 10^−4^ was applied identically to the cross-entropy and focal-loss configurations. As with the other conditions, every baseline was evaluated under 5-fold cross-validation, crossed with 5 random seeds.

### 2.4. Grad-CAM++ Visualization for Verifying Feature-Focused Learning

To interpret the decision-making process of the deep learning model, the Grad-CAM++ technique [31], as defined in Equation (3), was used. This visualization was employed to verify whether the application of focal loss enables the model to mitigate size-based shortcut learning and focus on important features of objects. Grad-CAM++ visualization was performed on both the model employing cross-entropy loss and the model applying focal loss on the same background-removed dataset, and activation regions were compared through color maps to confirm how the change in loss function affects the model’s decision-making basis. The progression toward red indicates that the model assigns higher weights in the decision-making process.

(3) $$L_{Grad-CAM++}^{c}=ReLu(\sum_{k}w_{k}^{C}A^{k})$$

Beyond qualitative visualization, two quantitative metrics were computed to objectively evaluate whether the activation regions overlap with the actual object regions. The Pointing Game accuracy [32] measures whether the location of the maximum activation in each Grad-CAM++ heatmap falls within the ground-truth object mask. A hit is recorded when this condition is satisfied, and the Pointing Game accuracy is reported as the proportion of hits over all evaluated images. The Intersection-over-Union (IoU) measures the spatial overlap between the high-activation region of each Grad-CAM++ heatmap and the ground-truth object mask, defined as in Equation (4).

(4) $$IoU=\frac{\left|A_{cam}\cap A_{obj}\right|}{\left|A_{cam}\cup A_{obj}\right|}$$

Here, $A_{obj}$ denotes the ground-truth object mask obtained from the rembg-based background removal pipeline used during preprocessing, and $A_{cam}$ denotes the binary mask obtained by thresholding the normalized Grad-CAM++ heatmap at 0.5. Both metrics were computed on the internal test set and the external Herb-X test set, and the cross-entropy and focal loss models were compared in a paired manner per image. To examine the cross-acquisition stability of attention, the change in each metric from the internal to the external test set was additionally analyzed.

### 2.5. Model Evaluation

The following standard evaluation metrics were employed to quantitatively assess the classification performance of the proposed model. All metrics were computed using an independent test dataset, with accuracy, as defined in Equation (5), adopted as the primary indicator representing the proportion of correctly classified samples among all test instances.

(5) $$Accuracy=\frac{TP+TN}{TP+TN+FP+FN}$$

Specifically, TP (True Positive) denotes cases in which *Z. jujuba* was correctly classified, TN (True Negative) denotes cases in which *Z. mauritiana* was correctly classified, and FP (False Positive) and FN (False Negative) represent the respective types of misclassifications. To comprehensively evaluate class-wise performance, additional metrics, including Precision, Recall, and F1-score, were analyzed using Equations (6)–(8).

(6) $$Precision=\frac{TP}{TP+FP}$$

(7) $$Recall=\frac{TP}{TP+FN}$$

(8) $$F1-score=2\times \frac{Precision\times Recall}{Precision+Recall}$$

Furthermore, a confusion matrix was generated to visualize the classification outcomes.

To ensure the statistical reliability of the comparisons among loss functions, dataset configurations, and architectures, all reported metrics correspond to the mean ± standard deviation across the 5-fold × 5-seed ensemble.

### 2.6. Hardware and Software Experimental Environment

All experiments were conducted on a workstation equipped with an NVIDIA RTX A5000 graphics processing unit and dual Intel Xeon Gold 6426Y processors (Intel Corporation, Santa Clara, CA, USA) (64-bit architecture, 32 cores/64 threads, maximum operating frequency of 4.10 GHz). The software environment was configured with Python 3.13, PyTorch 2.4.0, and CUDA 12.1.

## 3. Results

### 3.1. Analyzing Object Size Differences in the Dataset

**Figure 6:**
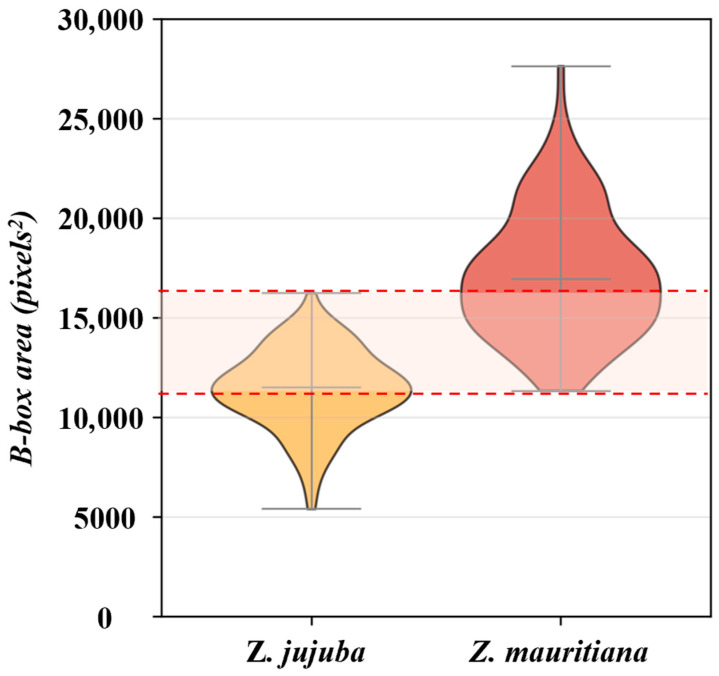
Bounding box area distributions of *Z. jujuba* and *Z. mauritiana*. The shaded background indicates the sample-level overlap range where the size distributions of the two species coexist.

To verify the object-size discrepancy between *Z. jujuba* and *Z. mauritiana* within the dataset, the bounding box areas of all specimens in the external test set were measured (Figure 6).

Within the external test set, the mean bounding box areas were 11,413 ± 1972 pixels^2^ for *Z. jujuba* and 17,132 ± 3175 pixels^2^ for *Z. mauritiana*, with *Z. mauritiana* being 50.1% larger on average. Despite the pronounced mean-level difference, the size distributions of the two species overlapped substantially at the sample level. Within the overlap range of 11,336–16,226 pixels^2^, 45.0% of *Z. mauritiana* and 53.0% of *Z. jujuba* samples coexisted.

### 3.2. Performance Evaluation Across Different Loss Functions

#### 3.2.1. Cross-Entropy Loss on the Internal Test Set

First, performance was evaluated after training on the background-removed dataset using cross-entropy loss. The CNN model trained on the background-removed dataset achieved a mean accuracy of 98.26 ± 2.95%, while the corresponding ResNet-50 model achieved 98.84 ± 1.20% (Table 1). The CNN model trained on the Upsizing dataset achieved a mean accuracy of 98.64 ± 1.16%, and the corresponding ResNet-50 model achieved 99.80 ± 0.42%. The CNN model trained on the Downsizing dataset achieved a mean accuracy of 98.94 ± 0.64%, and the corresponding ResNet-50 model achieved 99.52 ± 0.73%. Across the six cross-entropy configurations, mean accuracies on the internal test set ranged from 98.26% to 99.80%. Detailed class-wise precision and recall values are reported in Table 1.

#### 3.2.2. Focal Loss on the Internal Test Set

**Figure 7:**
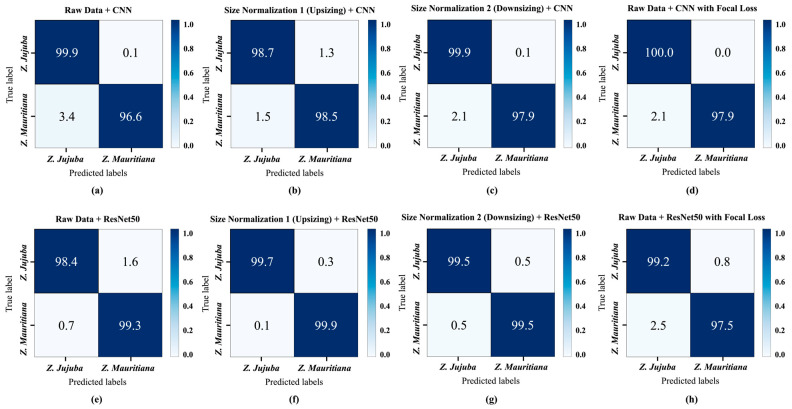
Confusion matrices on the internal test set, averaged across 5-fold × 5-seed: (**a**) CNN, CE, background-removed; (**b**) CNN, CE, Upsizing; (**c**) CNN, CE, Downsizing; (**d**) CNN, FL, background-removed; (**e**) ResNet-50, CE, background-removed; (**f**) ResNet-50, CE, Upsizing; (**g**) ResNet-50, CE, Downsizing; (**h**) ResNet-50, FL, background-removed.

The performance of the model applying focal loss was then evaluated under the same internal test conditions. The CNN model trained with focal loss on the background-removed dataset achieved a mean accuracy of 98.99 ± 0.78%, and the corresponding ResNet-50 model achieved 98.38 ± 2.21% (Table 1). Compared with the corresponding cross-entropy baselines on the same background-removed dataset, focal loss yielded an increase of 0.73 percentage points (pp) in mean accuracy for the CNN model and a decrease of 0.46 pp for the ResNet-50 model. Across the eight conditions evaluated on the internal test set, the focal loss model on the CNN exhibited the smallest cross-seed standard deviation. The corresponding confusion matrices for all configurations on the internal test set are shown in Figure 7.

### 3.3. Performance Evaluation on the External Dataset (Herb-X)

Based on the validation results reported in Section 3.2, the same set of trained models was further evaluated on the external dataset acquired with the Herb-X described in Section 2.1. The external dataset was constructed from a new set of herbal medicine specimens, captured under different imaging conditions from those used for the internal dataset, in order to test model performance under distribution shift.

#### 3.3.1. Cross-Entropy Loss on the External Test Set

Performance was first evaluated on the external dataset using the model trained with cross-entropy loss. The CNN model trained on the background-removed dataset achieved a mean accuracy of 82.08 ± 10.97%, while the corresponding ResNet-50 model achieved 82.69 ± 12.71% (Table 2). The CNN model trained on the Upsizing dataset achieved a mean accuracy of 84.17 ± 10.15%, and the corresponding ResNet-50 model achieved 87.17 ± 9.14%. The CNN model trained on the Downsizing dataset achieved a mean accuracy of 88.94 ± 6.76%, and the corresponding ResNet-50 model achieved 83.19 ± 13.47%. Across the six cross-entropy configurations, mean accuracies on the external test set ranged from 82.08% to 88.94%, with cross-seed standard deviations ranging from 6.76% to 13.47%. Detailed class-wise precision and recall values are reported in Table 2.

#### 3.3.2. Focal Loss on the External Test Set

**Figure 8:**
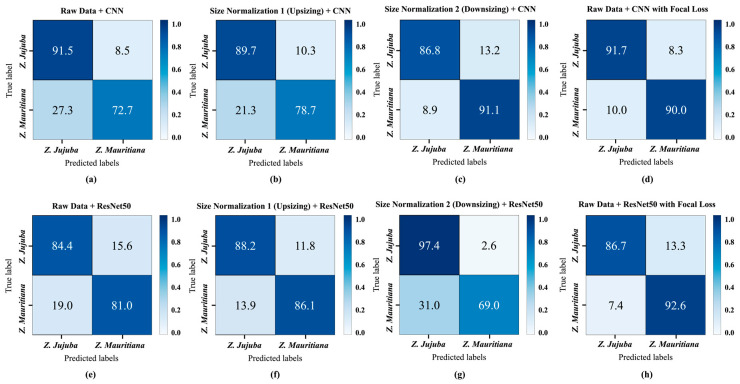
Confusion matrices on the Herb-X test set, averaged across 5-fold × 5-seed: (**a**) CNN, CE, background-removed; (**b**) CNN, CE, Upsizing; (**c**) CNN, CE, Downsizing; (**d**) CNN, FL, background-removed; (**e**) ResNet-50, CE, background-removed; (**f**) ResNet-50, CE, Upsizing; (**g**) ResNet-50, CE, Downsizing; (**h**) ResNet-50, FL, background-removed.

The model trained with focal loss on the background-removed dataset was then evaluated on the external test set. The CNN model achieved a mean accuracy of 90.88 ± 2.71%, and the corresponding ResNet-50 model achieved 89.63 ± 6.89% (Table 2). Compared with the corresponding cross-entropy baselines on the same background-removed dataset, focal loss yielded an increase of 8.80 pp in mean accuracy for the CNN model and an increase of 6.94 pp for the ResNet-50 model. Compared with the size-normalized cross-entropy configurations, the focal loss model on the CNN exceeded the corresponding Upsizing model by 6.71 pp and the corresponding Downsizing model by 1.94 pp. The focal loss model on ResNet-50 exceeded the corresponding Upsizing model by 2.46 pp and the corresponding Downsizing model by 6.44 pp. Across the eight conditions evaluated on the external test set, the focal loss model on the CNN exhibited the smallest cross-seed standard deviation (2.71%). The figure representing these results as a confusion matrix is shown in Figure 8.

#### 3.3.3. Internal–External Generalization Gap

The internal-to-external accuracy difference was computed for each of the eight model configurations to quantify generalization under distribution shift (Table 1 and Table 2). For the CNN, the cross-entropy models yielded gaps of 16.18 pp on the background-removed dataset, 14.47 pp on the Upsizing dataset, and 10.00 pp on the Downsizing dataset, whereas the focal loss model on the background-removed dataset yielded a gap of 8.11 pp. For ResNet-50, the cross-entropy models yielded gaps of 16.15 pp on the background-removed dataset, 12.63 pp on the Upsizing dataset, and 16.33 pp on the Downsizing dataset, whereas the focal loss model on the background-removed dataset yielded a gap of 8.75 pp. Across all eight configurations, the focal loss models on the background-removed dataset exhibited the smallest internal-to-external gaps in both architectures.

### 3.4. Misclassification Analysis on External Dataset

To examine whether the residual errors of the CNN models are systematically associated with object size, the bounding-box areas of all misclassified specimens were aggregated across the 5-fold × 5-seed ensemble on the external test set. Two error directions were analyzed, namely *Z. jujuba* misclassified as *Z. mauritiana* and *Z. mauritiana* misclassified as *Z. jujuba*. Reference statistics from the training set are reported for context, with mean bounding box areas of 11,413 pixels^2^ for *Z. jujuba* and 17,132 pixels^2^ for *Z. mauritiana*.

On the external test set, the cross-entropy CNN exhibited a misclassification rate of 17.92 ± 10.97% across the 25 independent runs, and the focal loss CNN exhibited a rate of 9.12 ± 2.71%. For the cross-entropy CNN, 24 of 25 models exhibited *Z. jujuba* misclassified as *Z. mauritiana* with a mean bounding-box area of 12,764 ± 1967 pixels^2^, and 23 of 25 models exhibited *Z. mauritiana* misclassified as *Z. jujuba* with a mean bounding-box area of 15,407 ± 872 pixels^2^. For the focal loss CNN, all 25 models exhibited *Z. jujuba* misclassified as *Z. mauritiana* with a mean bounding-box area of 11,837 ± 1054 pixels^2^, and all 25 models exhibited *Z. mauritiana* misclassified as *Z. jujuba* with a mean bounding-box area of 15,270 ± 1083 pixels^2^.

Across both loss functions, the mean bounding-box areas of misclassified specimens (11,837–15,407 pixels^2^) fell entirely within the size-overlap zone of 11,336–16,226 pixels^2^ previously identified between the two species. *Z. jujuba* specimens misclassified as *Z. mauritiana* exceeded the external-test *Z. jujuba* mean of 11,413 pixels^2^, while *Z. mauritiana* specimens misclassified as *Z. jujuba* fell below the external-test *Z. mauritiana* mean of 17,132 pixels^2^. This convergence indicates that residual errors arose predominantly from larger-than-typical *Z. jujuba* and smaller-than-typical *Z. mauritiana* specimens, whose object sizes are inherently ambiguous between classes.

### 3.5. Grad-CAM++-Based Visual Interpretation of Model Feature Focus

**Figure 9:**
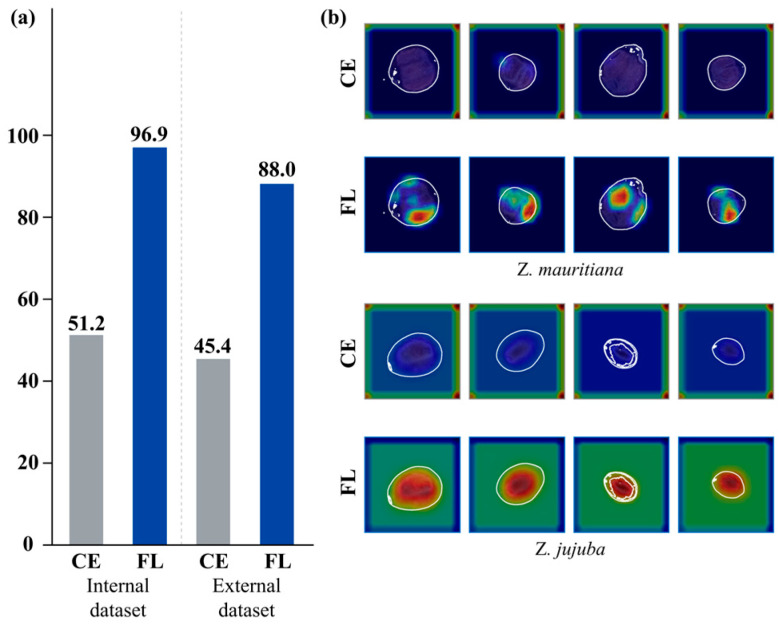
Quantitative and qualitative evaluation of Grad-CAM++ activation regions on the internal and external test sets: (**a**) Pointing Game accuracy comparison between the cross-entropy (CE) and focal loss (FL) CNN models; (**b**) Representative Grad-CAM++ heatmaps for *Z. mauritiana* (top two rows) and *Z. jujuba* (bottom two rows) on the external test set. In (**b**), red indicates higher activation, while blue indicates lower activation.

To visualize that the CNN applying focal loss focuses on detailed morphological features of objects rather than overfitting to object size, a subset of the test data was visualized using Grad-CAM++ (Figure 9). Analysis results revealed differences between the cross-entropy CNN model and the focal loss CNN model. In the cross-entropy model, activation tended to be distributed across the entire object region, with weights assigned to overall shape or contour rather than localized features. In contrast, in the focal loss model, higher weights were assigned to localized and specific features of objects, resulting in concentrated color maps. The activation regions were compared with the morphological characteristics of *Z. jujuba* and *Z. mauritiana* described in the Sensory Evaluation Manual of the Ministry of Food and Drug Safety of the Republic of Korea, and the focal loss model was found to focus on the scale-like surface patterns of *Z. mauritiana* and the raised ridge structures and concave linear hilum of *Z. jujuba*.

Beyond qualitative visualization, two quantitative metrics, namely Pointing Game accuracy and Intersection-over-Union (IoU), were computed across both the internal test set and the external Herb-X test set to objectively evaluate whether the activation regions overlap with the actual object regions (Table 3 and Figure 9).

On the internal test set, the cross-entropy CNN model achieved a Pointing Game accuracy of 51.22%, and the focal loss CNN model achieved 96.94%. The mean IoU was 0.1525 ± 0.1784 for the cross-entropy model and 0.3592 ± 0.2774 for the focal loss model.

On the external Herb-X test set, the cross-entropy CNN model achieved a Pointing Game accuracy of 45.42%, and the focal loss CNN model achieved 88.02%. The mean IoU was 0.1088 ± 0.1608 for the cross-entropy model and 0.3709 ± 0.2598 for the focal loss model.

The cross-acquisition stability of attention was further evaluated by comparing each metric between the internal and external test sets. The Pointing Game accuracy of the focal loss model decreased from 96.94% on the internal test set to 88.02% on the external test set, a difference of 8.92 pp, while the cross-entropy model showed a corresponding decrease from 51.22% to 45.42%, a difference of 5.80 pp. The mean IoU of the focal loss model was 0.3592 on the internal test set and 0.3709 on the external test set, while that of the cross-entropy model was 0.1525 on the internal test set and 0.1088 on the external test set.

### 3.6. Per-Sample Mechanistic Verification of Focal Loss

To examine how focal loss redistributes the per-sample training signal at the level of individual specimens, the modulating factor and the sample loss were computed on the external test set for both the cross-entropy and focal loss CNN models trained on the background-removed dataset. Specimens of each class were stratified into a shortcut-supportive subgroup and a shortcut-violating subgroup using the per-class median bounding-box area in the external test set as the splitting threshold. The shortcut-supportive subgroup comprises specimens whose object size aligns with the dataset’s class–size correlation, namely large *Z. mauritiana* and small *Z. jujuba*; the shortcut-violating subgroup comprises the opposite, namely small *Z. mauritiana* and large *Z. jujuba*.

#### 3.6.1. Sample Modulating Factor of Focal Loss

The modulating factor of the focal loss model was compared between the shortcut-supportive and shortcut-violating subgroups within each class, with results summarized in Table 4. For *Z. mauritiana*, the mean modulating factor was 0.0216 ± 0.0880 in the shortcut-supportive subgroup and 0.0914 ± 0.1690 in the shortcut-violating subgroup, corresponding to a 4.23-fold difference in favor of the violating subgroup. For *Z. jujuba*, the mean modulating factor was 0.0381 ± 0.1089 in the shortcut-supportive subgroup and 0.0762 ± 0.1873 in the shortcut-violating subgroup, corresponding to a 2.00-fold difference, again in favor of the violating subgroup.

#### 3.6.2. Sample Cross-Entropy Loss After Training

The per-sample cross-entropy loss after training was compared between the two models on the same stratified subgroups (Table 4). For *Z. mauritiana*, the focal loss model substantially reduced the per-sample loss in both subgroups: the mean loss decreased from 0.396 ± 0.566 to 0.076 ± 0.231 in the shortcut-supportive subgroup, an 81% reduction, and from 1.268 ± 1.257 to 0.296 ± 0.421 in the shortcut-violating subgroup, a 77% reduction. For *Z. jujuba*, the per-sample loss was higher under focal loss than under cross-entropy in both subgroups: the mean loss increased from 0.024 ± 0.067 to 0.171 ± 0.280 in the shortcut-supportive subgroup and from 0.114 ± 0.243 to 0.279 ± 0.541 in the shortcut-violating subgroup. However, this increase did not reflect a degradation in classification, as the focal loss model still classified *Z. jujuba* samples with high accuracy on the external test set.

### 3.7. Comparison with Alternative Baseline Methods

To verify that focal loss is not merely outperforming a vanilla cross-entropy baseline but also outperforming alternative interventions commonly used to address generalization or class-imbalance issues, three additional baseline methods, namely Class-Balanced Loss, Label Smoothing, and Scale Jittering, were evaluated on the CNN architecture trained on the background-removed dataset. All five configurations, namely cross-entropy, focal loss, Class-Balanced Loss, Label Smoothing, and Scale Jittering, were evaluated under the same 5-fold × 5-seed protocol used throughout this study (Table 5).

On the internal test set, the cross-entropy CNN model achieved a mean accuracy of 98.26 ± 2.95%, the focal loss CNN model achieved 98.99 ± 0.78%, the Class-Balanced Loss CNN model achieved 97.83 ± 3.40%, the Label Smoothing CNN model achieved 97.79 ± 3.72%, and the Scale Jittering CNN model achieved 99.08 ± 0.63%.

On the external Herb-X test set, the cross-entropy CNN model achieved a mean accuracy of 82.08 ± 10.97%, the focal loss CNN model achieved 90.88 ± 2.71%, the Class-Balanced Loss CNN model achieved 86.98 ± 7.00%, the Label Smoothing CNN model achieved 82.79 ± 11.25%, and the Scale Jittering CNN model achieved 82.13 ± 9.72%. The focal loss CNN model exhibited the highest mean external accuracy and the smallest cross-seed standard deviation.

The internal-to-external accuracy difference was computed for each of the five configurations to quantify generalization under distribution shift. The cross-entropy CNN model showed an internal-to-external gap of 16.18 pp, the focal loss CNN model showed a gap of 8.11 pp, the Class-Balanced Loss CNN model showed a gap of 10.85 pp, the Label Smoothing CNN model showed a gap of 14.99 pp, and the Scale Jittering CNN model showed a gap of 16.95 pp. Across the five configurations, the focal loss CNN model exhibited the smallest internal-to-external gap.

## 4. Discussion

### 4.1. Size-Based Shortcut Learning in Herbal Medicine Classification

Deep learning models trained on limited data often exhibit shortcut learning, in which the model relies on the most easily learnable features to achieve immediate high performance rather than learning intrinsic discriminative characteristics. Since herbal medicines can exhibit considerable variation even within the same species, depending on factors such as geographic origin, harvest season, and processing methods, developing field-applicable models requires the learning of intrinsic and invariant features rather than superficial characteristics present only in specific datasets.

First, the absolute bounding box areas differed markedly between the training set and the external test set. This disparity is attributable to differences in image-acquisition conditions, including camera specifications, shooting distance, and field of view, between the smartphone-captured training set and the Herb-X embedded-camera external test set [19]. This observation indicates that absolute pixel-level size is a highly variable feature that fluctuates with the acquisition setup, and that relying on it as a primary classification cue would inevitably compromise the external generalization performance of the model.

Second, although *Z. mauritiana* specimens were on average consistently and significantly larger than *Z. jujuba* specimens within each acquisition condition, the two species’ size distributions overlapped considerably at the sample level; in the external test set in particular, near-majority proportions of both classes fell within the overlap range. This demonstrates that the observed mean-level size ordering does not represent a universal biological property, in that individual *Z. jujuba* specimens can exceed individual *Z. mauritiana* specimens in size, and that size alone cannot reliably discriminate the two species at the individual-specimen level. These two constraints have direct implications for whether object size can legitimately serve as a classification feature. One methodological approach would be to incorporate size explicitly as a predictor rather than removing it through preprocessing; however, given the combined effects of acquisition-dependent scale variability and sample-level distributional overlap inherent in this dataset, size itself behaves as a variable feature. A model trained to discriminate by size during training would suffer performance degradation when deployed under different image-acquisition environments, because the pixel-level size distribution of each class shifts unpredictably with the acquisition setup.

Therefore, suppressing size-based shortcuts is a prerequisite for building a model that remains reliable in real-world environments beyond the training setting. These findings support the need for the model to focus on the unique and intrinsic morphological features that distinguish *Z. jujuba* from *Z. mauritiana*, such as surface texture, ridge structure, and hilum morphology, rather than relying on variable features such as size. At the same time, the consistent mean-level size bias embedded in the data provides an empirical basis for why CNN models trained on this dataset are prone to size-based shortcut learning, and justifies the training strategy adopted in the present study to explicitly suppress such behavior.

The internal evaluation of the present study showed that all CNN and ResNet-50 model configurations achieved comparably high accuracies of at least 98% regardless of architecture, dataset configuration, or loss function (Table 1). When the same models were evaluated on the external Herb-X dataset, mean accuracies decreased to a range of 82.08% to 88.94% across the six cross-entropy configurations (Table 2), and the corresponding internal-to-external gaps reached up to 16.18 pp for the CNN and 16.33 pp for ResNet-50.

Direct evidence that this gap is mediated by object size comes from the misclassification analysis on the external test set (Section 3.4). Across the 25 cross-entropy CNN models trained on the background-removed dataset, *Z. mauritiana* specimens misclassified as Z. jujuba had a mean bounding-box area of 15,407 ± 872 pixels^2^, below the external *Z. mauritiana* mean of 17,132 pixels^2^, while *Z. jujuba* specimens misclassified as *Z. mauritiana* had a mean of 12,764 ± 1967 pixels^2^, above the external *Z. jujuba* mean of 11,413 pixels^2^. Both subsets fell within the inter-class overlap range (11,336–16,226 pixels^2^), where size alone cannot reliably discriminate the two species. Thus, on the external test set, the residual errors of the cross-entropy CNN are concentrated on specimens whose object size violates the dataset’s class–size correlation, demonstrating that size-based shortcut learning has occurred.

### 4.2. Focal Loss as a Solution to Size-Based Shortcut Learning

#### 4.2.1. Focal Loss Preventing Size-Based Shortcut Learning Through Loss Analysis

The per-sample weighting analysis on the external test set was used to confirm how focal loss prevents the model from relying on object size, with results summarized in Table 4. To examine which specimens received larger weights, the external specimens were divided into shortcut-supportive and shortcut-violating subgroups within each class based on bounding box area. The shortcut-supportive subgroup comprised specimens whose size matched the typical pattern of the training data, namely large *Z. mauritiana* and small *Z. jujuba*, whereas the shortcut-violating subgroup comprised specimens whose size differed from this pattern, namely small *Z. mauritiana* and large *Z. jujuba*. The shortcut-supportive subgroup can, in principle, be classified correctly by relying on object size alone, whereas the shortcut-violating subgroup requires the model to use morphological features beyond size.

The focal loss model assigned much higher weights to specimens in the shortcut-violating subgroup. For *Z. mauritiana*, the mean weight was 0.0914 ± 0.1690 in the shortcut-violating subgroup and 0.0216 ± 0.0880 in the shortcut-supportive subgroup, a 4.23-fold difference. For *Z. jujuba*, the mean weight was 0.0762 ± 0.1873 in the shortcut-violating subgroup and 0.0381 ± 0.1089 in the shortcut-supportive subgroup, a 2.00-fold difference. In other words, during training, the focal loss model spent most of its learning effort on specimens whose object size could not serve as a shortcut to the correct class. The downstream consequence of this redistribution is observed even more clearly in the sample loss values measured after training. For small *Z. mauritiana* specimens, the mean loss decreased from 1.268 ± 1.257 under cross-entropy to 0.296 ± 0.421 under focal loss, a 77% reduction.

#### 4.2.2. Improved Generalization Under Distribution Shift

Across all eight CNN and ResNet-50 configurations, the focal loss models trained on the background-removed dataset exhibited the smallest internal-to-external gaps in both architectures, namely 8.11 pp for the CNN and 8.75 pp for ResNet-50, compared with 16.18 pp and 16.15 pp for the corresponding cross-entropy baselines. This represents a halving of the generalization gap with no architectural change and no preprocessing-based size normalization. Notably, the cross-seed variability of the focal loss CNN model on the external test set was the smallest among all eight configurations, with a standard deviation of 2.71%, indicating that the gap reduction is reproducible across random initializations rather than being an artifact of a single fortunate seed.

The fact that this gap reduction is observed in both the custom CNN and ResNet-50 supports the interpretation that the effect arises from the loss function itself rather than from the specific inductive biases of either architecture. Equally importantly, applying focal loss alone, without size normalization, yielded a smaller external-internal gap than cross-entropy combined with explicit size normalization. The focal loss CNN model showed a gap of 8.11 pp, whereas the size-normalized cross-entropy CNN configurations showed gaps ranging from 10.00 to 14.47 pp. In other words, modifying the sample weighting of the gradient is more effective at producing a size-invariant model than rescaling the input pixels to remove size differences before training, while also obviating the need for a size-normalization step at deployment.

Several practical advantages of this loss-function-level intervention deserve emphasis. First, focal loss requires only a single change to the training objective and introduces no additional inference-time cost, in contrast to size normalization, which requires a separate preprocessing pipeline that must be reproduced at every deployment site to ensure consistent input scaling. Second, focal loss preserves the original spatial information of the input image, whereas size normalization actively removes differential scale information that may carry residual discriminative value. Third, the standard deviation of the focal loss model on the external test set was 2.71%, substantially smaller than the 6.76 to 13.47% range observed for size-normalized cross-entropy configurations, suggesting that the focal loss model is not only more accurate on average but also more stable across runs, a property that is important for any application requiring consistent performance.

### 4.3. Is Focal Loss the Only Solution for Size-Based Shortcut Learning?

To verify whether the improvement of focal loss could be obtained by other commonly used methods, three alternative baselines, namely Class-Balanced Loss, Label Smoothing, and Scale Jittering, were evaluated on the CNN architecture under the same protocol, with results summarized in Table 5. The Class-Balanced Loss CNN model achieved a mean external accuracy of 86.98 ± 7.00% and an internal-to-external gap of 10.85 pp, an improvement over the cross-entropy baseline that did not reach focal loss. The Label Smoothing CNN model achieved a mean external accuracy of 82.79 ± 11.25% and an internal-to-external gap of 14.99 pp, with little difference from the cross-entropy baseline. This indicates that softening the target distribution alone is not sufficient to redirect the model away from the size cue. The Scale Jittering CNN model achieved the highest mean internal accuracy among the five configurations at 99.08 ± 0.63%, but the lowest mean external accuracy at 82.13 ± 9.72% and the largest internal-to-external gap at 16.95 pp. This appears to be because rescaling each specimen broadens the size distribution within each class but preserves the underlying class–size correlation, so the model can continue to use size as a shortcut even after augmentation.

A noteworthy observation is that Class-Balanced Loss achieved the second-highest external accuracy after focal loss. This suggests that re-weighting the loss contribution of individual samples is an effective approach to suppressing the size-based shortcut. The internal-to-external gaps across the five configurations, namely 8.11 pp for focal loss, 10.85 pp for Class-Balanced Loss, 14.99 pp for Label Smoothing, 16.18 pp for cross-entropy, and 16.95 pp for Scale Jittering, demonstrate that focal loss provides the strongest reduction in the size-based shortcut among the methods tested.

### 4.4. Grad-CAM++ Visualization of Size-Based Shortcut Learning Prevention via Focal Loss

Grad-CAM++ analysis revealed that the cross-entropy CNN model distributed its activation across the entire object region, with weights placed on the overall shape and contour. In contrast, the focal loss CNN model concentrated its activation on the localized morphological features described above, namely the scale-like surface patterns of *Z. mauritiana* and the raised ridge structures of *Z. jujuba*. These results indicate that the focal loss model relies on the morphological features that genuinely distinguish the two species rather than on the size cue.

On the internal test set, the focal loss CNN model achieved a Pointing Game accuracy of 96.94% and a mean IoU of 0.3592 ± 0.2774, indicating that it attended to the actual object regions much more accurately than the cross-entropy CNN model. On the external Herb-X test set, the focal loss CNN model achieved a Pointing Game accuracy of 88.02% and a mean IoU of 0.3709 ± 0.2598, indicating that it consistently attended to the morphological features of the specimens despite changes in imaging device and specimens. In contrast, the cross-entropy CNN model showed a decrease in both Pointing Game accuracy and mean IoU between the two test sets, suggesting that it relies on features dependent on the specific imaging conditions of the training set.

### 4.5. Limitations and Future Directions

This study has several limitations. First, the possibility of other forms of shortcut learning beyond size, such as background color, lighting conditions, and shadow patterns, cannot be completely ruled out. In particular, RGB image-based classification models possess an inherent limitation in that classification performance can degrade when specimens are photographed under diverse environmental conditions or are physically damaged. To address this limitation, it is necessary to develop approaches that integrate chemical composition analysis techniques such as Hyperspectral Imaging or Near-Infrared Spectroscopy, combining morphological information with spectral information. Through this integration, more practical models with improved classification accuracy and greater robustness against various external confounding factors are expected to be developed.

Second, this study was conducted with a limited dataset of *Z. jujuba* and *Z. mauritiana* collected from a single geographic origin during the same period. Given the nature of herbal medicines, considerable variability exists in morphological and chemical characteristics depending on geographic origin, collection period, processing methods, and storage conditions. For practical deployment, the model must demonstrate robust generalization beyond controlled experimental conditions, encompassing the diverse and often unpredictable variations inherent to commercial distribution, such as inter-supplier differences, lot-to-lot variation, and physical alterations during packaging, transportation, and storage. These factors are typically underrepresented in curated research datasets. Future work should therefore prioritize the construction of large-scale datasets covering diverse geographic origins and collection periods, alongside systematic evaluation using distribution-stage samples obtained at scale from diverse channels and sources.

Third, the Herb-X was designed to maintain consistent imaging conditions through fixed camera-to-specimen geometry, controlled LED illumination, and black acrylic walls that block external ambient light. Nevertheless, its performance may still be affected by real-world factors such as ambient lighting at deployment sites and variability in user operation, which were not systematically evaluated in the present study. Systematic assessment of the device under diverse field conditions, including varied ambient lighting, deployment sites such as inspection centers, wholesale markets, and customs facilities, and a range of user-operation scenarios, represents an important direction for future work. Such evaluation will be essential for validating the practical reliability of the Herb-X-based inspection workflow across the full spectrum of real-world deployment contexts.

In addition, extending the framework to other herbal medicines prone to size-based shortcut learning represents a promising direction. Achieving clinically reliable robustness through these advancements would support the automation and standardization of herbal medicine quality control.

## 5. Conclusions

The present study investigated size-based shortcut learning in CNN-based classification of *Z. jujuba* and *Z. mauritiana*, and proposed focal loss as a loss-function-level intervention to address it. By comparing eight model configurations across two architectures, namely a custom CNN and ResNet-50, three dataset configurations, namely background-removed, Upsizing, and Downsizing, and two loss functions, namely cross-entropy and focal loss, the present study confirmed that size-based shortcut learning occurs in this task. The focal loss model achieved the smallest internal-to-external generalization gap of 8.11 percentage points, demonstrating that it is effective at resolving the performance degradation on the external dataset that arises from size-based shortcut learning.

Grad-CAM++ analysis and loss analysis confirmed that focal loss assigns 4.23-fold higher sample weights to *Z. mauritiana* specimens whose object size differs from the typical pattern of the training data, and concentrates the model’s attention on the morphological features that genuinely distinguish the two species, namely the scale-like surface patterns of *Z. mauritiana* and the raised ridge structures of *Z. jujuba*. Comparison with three alternative baseline methods, namely Class-Balanced Loss, Label Smoothing, and Scale Jittering, further showed that focal loss provided the strongest reduction in the size-based shortcut among the methods tested.

Therefore, focal loss enabled the construction of a robust classification model generalizable to an external dataset by suppressing size-based shortcut learning without complex preprocessing or extensive data augmentation. The integration of this model with the Herb-X is expected to contribute to addressing the shortage of sensory inspection personnel and to the automation of quality control processes. Future application of diverse herbal medicines is anticipated to ultimately contribute to resolving authenticity problems, thereby benefiting consumer safety in the herbal medicine market.

## Figures and Tables

**Table 1:** Performance comparison of CNN and ResNet-50 models with cross-entropy loss and focal loss on the internal test set.

Architecture	Loss Function	Input Data	Class	Accuracy (%)	Precision (%)	Recall (%)	F1-Score (%)
CNN	Cross- entropy loss	Background-removed	*Z.* *j* *ujuba*	98.26 ± 2.95	97.00 ± 4.65	99.88 ± 0.36	98.36 ± 2.61
			*Z.* *m* *auritiana*		99.88 ± 0.37	96.64 ± 5.80	98.31 ± 3.38
		Upsizing	*Z.* *j* *ujuba*	98.64 ± 1.16	98.60 ± 1.54	98.73 ± 1.99	98.64 ± 1.15
			*Z.* *m* *auritiana*		98.75 ± 1.96	98.55 ± 1.62	98.63 ± 1.17
		Downsizing	*Z.* *j* *ujuba*	98.94 ± 0.64	98.01 ± 1.23	99.94 ± 0.30	98.96 ± 0.62
			*Z.* *m* *auritiana*		99.94 ± 0.31	97.94 ± 1.31	98.92 ± 0.66
	Focal loss	Background-removed	*Z.* *j* *ujuba*	98.99 ± 0.78	98.06 ± 1.44	100.00 ± 0.00	99.01 ± 0.74
			*Z.* *m* *auritiana*		100.00 ± 0.00	97.94 ± 1.61	98.96 ± 0.83
ResNet-50	Cross- entropy loss	Background-removed	*Z.* *j* *ujuba*	98.84 ± 1.20	99.30 ± 1.02	98.38 ± 2.59	98.82 ± 1.31
			*Z.* *m* *auritiana*		98.45 ± 2.43	99.30 ± 1.02	98.85 ± 1.22
		Upsizing	*Z.* *j* *ujuba*	99.80 ± 0.42	99.88 ± 0.29	99.73 ± 0.68	99.80 ± 0.41
			*Z.* *m* *auritiana*		99.72 ± 0.70	99.88 ± 0.29	99.80 ± 0.42
		Downsizing	*Z.* *j* *ujuba*	99.52 ± 0.73	99.52 ± 0.81	99.55 ± 1.26	99.53 ± 0.74
			*Z.* *m* *auritiana*		99.55 ± 1.24	99.51 ± 0.82	99.52 ± 0.73
	Focal loss	Background-removed	*Z.* *j* *ujuba*	98.38 ± 2.21	97.69 ± 3.31	99.22 ± 3.25	98.39 ± 2.26
			*Z.* *m* *auritiana*		99.33 ± 2.74	97.52 ± 3.60	98.36 ± 2.19

**Table 2:** Performance comparison of CNN and ResNet-50 models with cross-entropy loss and focal loss evaluated on the external dataset (Herb-X).

Architecture	Loss Function	Input Data	Class	Accuracy (%)	Precision (%)	Recall (%)	F1-Score (%)
CNN	Cross- entropy loss	Background-removed	*Z.* *j* *ujuba*	82.08 ± 10.97	80.19 ± 13.51	91.49 ± 14.72	83.56 ± 11.13
			*Z.* *m* *auritiana*		91.07 ± 10.88	72.70 ± 23.37	78.09 ± 18.26
		Upsizing	*Z.* *j* *ujuba*	84.17 ± 10.15	79.68 ± 19.88	89.68 ± 21.03	83.36 ± 18.38
			*Z.* *m* *auritiana*		92.20 ± 11.21	78.68 ± 18.31	82.61 ± 11.55
		Downsizing	*Z.* *j* *ujuba*	88.94 ± 6.76	92.51 ± 9.11	86.77 ± 10.10	88.72 ± 6.54
			*Z.* *m* *auritiana*		88.44 ± 6.86	91.11 ± 14.78	88.55 ± 9.49
	Focal loss	Background-removed	*Z.* *j* *ujuba*	90.88 ± 2.71	90.84 ± 6.09	91.73 ± 4.99	90.99 ± 2.33
			*Z.* *m* *auritiana*		92.04 ± 3.99	90.03 ± 7.88	90.68 ± 3.44
ResNet-50	Cross- entropy loss	Background-removed	*Z.* *j* *ujuba*	82.69 ± 12.71	86.30 ± 14.97	84.43 ± 18.61	82.64 ± 13.18
			*Z.* *m* *auritiana*		86.64 ± 11.60	82.69 ± 12.71	79.79 ± 21.51
		Upsizing	*Z.* *j* *ujuba*	87.17 ± 9.14	89.13 ± 11.52	88.21 ± 14.46	87.10 ± 9.71
			*Z.* *m* *auritiana*		90.08 ± 9.07	86.10 ± 18.87	85.98 ± 13.38
		Downsizing	*Z.* *j* *ujuba*	83.19 ± 13.47	79.30 ± 15.25	97.41 ± 2.89	86.41 ± 8.99
			*Z.* *m* *auritiana*		97.90 ± 2.70	69.04 ± 28.84	76.34 ± 24.45
	Focal loss	Background-removed	*Z.* *j* *ujuba*	89.63 ± 6.89	93.73 ± 8.70	86.65 ± 11.20	89.19 ± 7.15
			*Z.* *m* *auritiana*		88.59 ± 7.67	92.60 ± 11.20	89.49 ± 9.29

**Table 3:** Grad-CAM++ quantitative evaluation on the internal and external test sets, comparing the cross-entropy and focal loss CNN models on the background-removed dataset.

Test Set	Loss	Pointing Game (%)	IoU (Mean ± SD)
Internal	Cross-entropy	51.22	0.1525 ± 0.1784
	Focal loss	96.94	0.3592 ± 0.2774
External (Herb-X)	Cross-entropy	45.42	0.1088 ± 0.1608
	Focal loss	88.02	0.3709 ± 0.2598

**Table 4:** Sample loss and modulating factor analysis on the external test set, comparing the cross-entropy and focal loss CNN models. Specimens were stratified into shortcut-supportive and shortcut-violating subgroups based on bounding box area relative to the median size of each class, with shortcut-supportive corresponding to large samples for *Z. mauritiana* and small samples for *Z. jujuba*.

Class	Subgroup Label	FL Modulating Factor (Mean ± SD)	CE Loss (Mean ± SD)	FL (Mean ± SD)	$\mathrm{CE}\;p_{t}$	$\mathrm{FL}\;p_{t}$
*Z. jujuba*	Shortcut supportive	0.0381 ± 0.1089	0.024 ± 0.067	0.171 ± 0.280	0.979	0.866
	Shortcut violating	0.0762 ± 0.1873	0.114 ± 0.243	0.279 ± 0.541	0.911	0.822
*Z. mauritiana*	Shortcut supportive	0.0216 ± 0.0880	0.396 ± 0.566	0.076 ± 0.231	0.761	0.945
	Shortcut violating	0.0914 ± 0.1690	1.268 ± 1.257	0.296 ± 0.421	0.472	0.794

**Table 5:** Comparison of focal loss against alternative baseline methods on the CNN architecture trained on the background-removed dataset.

Method	Internal Accuracy (%)	External Accuracy (%)	Internal–External Gap (pp)
Cross-entropy	98.26 ± 2.95	82.08 ± 10.97	16.18
Focal loss	98.99 ± 0.78	90.88 ± 2.71	8.11
Class-Balanced Loss	97.83 ± 3.40	86.98 ± 7.00	10.85
Label Smoothing	97.79 ± 3.72	82.79 ± 11.25	14.99
Scale Jittering	99.08 ± 0.63	82.13 ± 9.72	16.95

## Data Availability

The datasets presented in this article are not readily available because the data are part of ongoing research with the National Institute for Korean Medicine Development. Requests to access the datasets should be directed to the corresponding author.

## Abbreviations

The following abbreviations are used in this manuscript:

AI	Artificial Intelligence
CNN	Convolutional Neural Network
Grad-CAM++	Gradient-weighted Class Activation Mapping++
HSI	Hyperspectral Imaging
LCD	Liquid Crystal Display
NIR	Near-Infrared Spectroscopy
RGB	Red, Green, and Blue
TLC	Thin-Layer Chromatography
XAI	Explainable Artificial Intelligence
